# Disseminated crusted scabies in an elderly patient

**DOI:** 10.1590/0037-8682-0348-2020

**Published:** 2020-12-21

**Authors:** Luís Felipe Teixeira Neumaier, Diego Chemello, Raíssa Massaia Londero Chemello

**Affiliations:** 1 Universidade Federal de Santa Maria, Departamento de Dermatologia, Santa Maria, RS, Brasil.; 2 Universidade Federal de Santa Maria, Departamento de Clínica Médica, Santa Maria, RS, Brasil.

A 66-year-old woman was referred to the emergency room for disseminated crusted cutaneous lesions. One year prior, she had presented with pruritus and erythematous papules in the retroauricular area that spread to the scalp, trunk, and limbs. Since the lesions persisted, a skin biopsy was performed three months before admission, and a diagnosis of parapsoriasis was suggested by a clinical physician. The patient was then started on methotrexate (20mg per week) and clobetasol cream. The lesions spread all over the body. Oral methylprednisolone (30mg per day for five days), antihistamines, and cephalexin were also initiated. Despite great improvement in the pruritus, the lesions continued to spread.

At the tertiary hospital, the patient presented with mental confusion and fever. Physical examination revealed disseminated greenish-gray, crusted lesions associated with skin fissures and erythema (Figure A). Direct microscopy of a skin scraping revealed scabies mites, and Norwegian scabies was diagnosed. Treatment with oral ivermectin and topical permethrin 5% was started. Since a secondary bacterial skin infection was probable, amoxicillin with clavulanic acid was administered for seven days. Close contacts received guidance and treatment as per guidelines^1^. Investigations for immunosuppression and occult neoplasms were negative. The patient was discharged with improvement of the lesions. A 15-day follow-up at the Dermatologic Clinic showed marked improvement (Figure B).

**FIGURE A:**
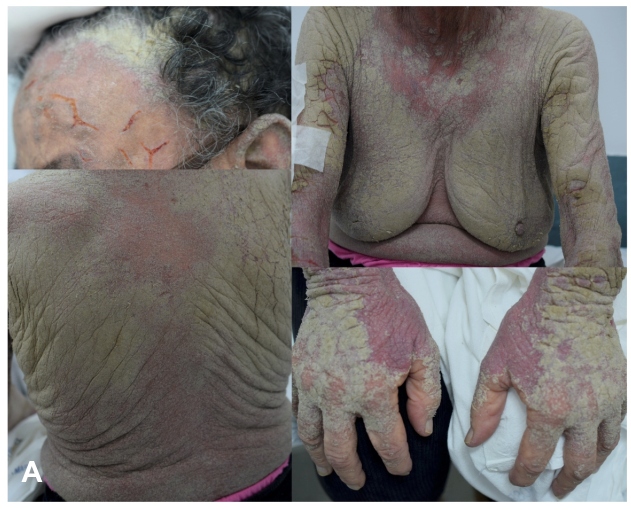
Disseminated greenish-gray crusted lesions associated with skin fissures and erythema.

**FIGURE B:**
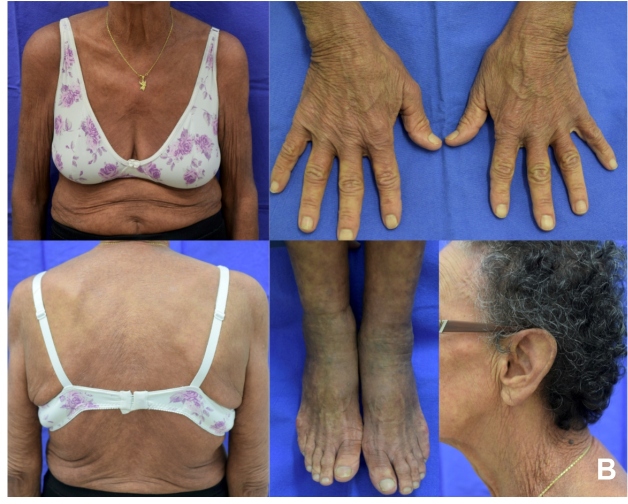
15-day follow-up at the Dermatologic Clinic showed marked improvement of cutaneous lesions.

Crusted scabies are characterized by hyperkeratotic lesions with cutaneous fissures and intense itching, which worsens at night^2^ ^,^ ^3^. In addition to exuberant lesions, this case highlights the importance of early diagnosis and treatment of scabies.
